# Water Filtered Infrared A and Visible Light (wIRA/VIS) Irradiation Reduces *Chlamydia trachomatis* Infectivity Independent of Targeted Cytokine Inhibition

**DOI:** 10.3389/fmicb.2018.02757

**Published:** 2018-11-15

**Authors:** Jasmin Kuratli, Theresa Pesch, Hanna Marti, Cory Ann Leonard, Christian Blenn, Paul Torgerson, Nicole Borel

**Affiliations:** ^1^Institute of Veterinary Pathology, Vetsuisse Faculty, University of Zurich, Zurich, Switzerland; ^2^Section of Veterinary Epidemiology, Vetsuisse Faculty, University of Zurich, Zurich, Switzerland

**Keywords:** *Chlamydia trachomatis*, Celastrol, Maraviroc, wIRA irradiation, wIRA/VIS, cytokine gene silencing

## Abstract

*Chlamydia trachomatis* is the major cause of infectious blindness and represents the most common bacterial sexually transmitted infection worldwide. Considering the potential side effects of antibiotic therapy and increasing threat of antibiotic resistance, alternative therapeutic strategies are needed. Previous studies showed that water filtered infrared A alone (wIRA) or in combination with visible light (wIRA/VIS) reduced *C. trachomatis* infectivity. Furthermore, wIRA/VIS irradiation led to secretion of pro-inflammatory cytokines similar to that observed upon *C. trachomatis* infection. We confirmed the results of previous studies, namely that cytokine secretion (IL-6, IL-8, and RANTES/CCL5) upon wIRA/VIS treatment, and the subsequent reduction of chlamydial infectivity, are independent of the addition of cycloheximide, a host protein synthesis inhibitor. Reproducible cytokine release upon irradiation indicated that cytokines might be involved in the anti-chlamydial mechanism of wIRA/VIS. This hypothesis was tested by inhibiting IL-6, IL-8, and RANTES secretion in *C. trachomatis* or mock-infected cells by gene silencing or pharmaceutical inhibition. Celastrol, a substance derived from *Trypterygium wilfordii*, used in traditional Chinese medicine and known for anti-cancer and anti-inflammatory effects, was used for IL-6 and IL-8 inhibition, while Maraviroc, a competitive CCR5 antagonist and anti-HIV drug, served as a RANTES/CCL5 inhibitor. HeLa cell cytotoxicity and impact on chlamydial morphology, size and inclusion number was evaluated upon increasing inhibitor concentration, and concentrations of 0.1 and 1 μM Celastrol and 10 and 20 μM Maraviroc were subsequently selected for irradiation experiments. Celastrol at any concentration reduced chlamydial infectivity, an effect only observed for 20 μM Maraviroc. Triple dose irradiation (24, 36, 40 hpi) significantly reduced chlamydial infectivity regardless of IL-6, IL-8, or RANTES/CCL5 gene silencing, Celastrol or Maraviroc treatment. Neither gene silencing nor pharmaceutical cytokine inhibition provoked the chlamydial stress response. The anti-chlamydial effect of wIRA/VIS is independent of cytokine inhibition under all conditions evaluated. Thus, factors other than host cell cytokines must be involved in the working mechanism of wIRA/VIS. This study gives a first insight into the working mechanism of wIRA/VIS in relation to an integral component of the host immune system and supports the potential of wIRA/VIS as a promising new tool for treatment in trachoma.

## Introduction

*Chlamydia trachomatis* represents both, the most common bacterial sexually transmitted infection and the major cause of infectious blindness, worldwide (Hu et al., 2013). The primary frontline antibiotic used to treat ocular chlamydial infection and prevent trachoma is azithromycin (Baneke, 2012), and current WHO recommendations constitute mass treatment with a single dose of azithromycin (WHO^∗^). Antibiotic treatment can cause unwanted side-effects, is expensive and, particularly when used improperly, may lead to antibiotic resistance. Although resistance to azithromycin in *C. trachomatis* has not yet been reported, resistance in *Streptococcus pneumoniae*, a major cause of morbidity and mortality worldwide, increased after azithromycin mass treatment (Ho et al., 2015). Experiences from genital *Chlamydia* control programs suggest that early antimicrobial treatment interferes with the development of protective immune responses, leading to the “arrested immunity hypothesis" (Brunham and Rekart, 2008). Moreover, a break in the normal chlamydial developmental cycle can result in long-term infection (Wyrick, 2010; Bavoil, 2014) and such infections can cause a cascade of ongoing inflammatory-induced sequelae resulting in scarring and fibrosis.

Chlamydiae are obligate intracellular bacteria with a complex developmental cycle comprising the infectious elementary body (EB) and the replicating reticulate body (RB). Under adverse environmental conditions, developing chlamydiae may enter a state referred to as persistence, more recently named the chlamydial stress response or the aberrant body (AB) phenotype (Wyrick, 2010; Bavoil, 2014; Leonard and Borel, 2014). This AB chlamydial form is more resistant, or even refractory, to antibiotic treatment *in vitro* and in animal models (Phillips-Campbell et al., 2014; Borel et al., 2016). Since antibiotic side effects (cardiac events/azithromycin – Lu et al., 2015), risk of development of antibiotic resistance (Suchland et al., 2009; Sandoz and Rockey, 2010; Vanrompay et al., 2017) and insufficient compliance during treatment (reviewed in Hammerschlag and Kohlhoff, 2012) represent serious drawbacks to current therapies, further therapeutic strategies are needed.

Water-filtered infrared A (wIRA) is infrared radiation with a spectrum of 780–1,400 nm, resulting from the light produced by a halogen bulb passing through a water cuvette to exclude wavelengths above 1,400 nm and through a black filter to block visible light (VIS; Jung et al., 2012). Various clinical trials have shown that wIRA alone and in combination with visible light (wIRA/VIS) improves acute and chronic wound healing processes (Hoffmann, 2007). Moreover, two studies showed that wIRA/VIS treatment of abdominal wounds, before or after surgery, not only improved wound healing and oxygen partial pressure, but also reduced the rate of wound infections (Hartel et al., 2006; Künzli et al., 2013).

Our initial studies (Marti et al., 2014) investigated whether wIRA/VIS irradiation can reduce the number of chlamydial inclusions, and therefore diminish recovery of both intra- and extracellular infectious EBs, in cells infected with either human (genital serovar E of *C. trachomatis*) or animal (*C. pecorum* originating from a porcine abortion) chlamydial strains. A single application of wIRA/VIS irradiation at 40 hours post-infection (hpi) led to a significant (up to 70%) reduction of infectivity in both strains of *chlamydiae*. Irradiation of host cells alone (HeLa or Vero) neither affected cell viability nor induced molecular markers of cytotoxicity (Marti et al., 2014). A triple application of irradiation (24, 36, 40 hpi) during the course of chlamydial infection further reduced chlamydial inclusion frequency in HeLa cells without inducing the unfavorable chlamydial persistence/chlamydial stress response. Quantitative analysis of cytokine and chemokine levels in supernatants of cell cultures subjected to triple irradiation revealed the release of pro-inflammatory cytokines and chemokines upon irradiation or infection alone, or in combination (Marti et al., 2014).

We carried out a follow-up study (Marti et al., 2015), which investigated factors influencing the effect of wIRA/VIS on acute chlamydial infection, namely the impact of temperature, irradiation intensity, infectious dose and the efficacy of the VIS component. Our findings demonstrated that thermal as well as non-thermal effects of wIRA/VIS contribute to the inhibition of acute chlamydial infection. Additionally, VIS enhanced the inhibitory effect of wIRA on extracellular EBs, but the effect of irradiation was not influenced by chlamydial infection dose. The infectivity of mature chlamydial inclusions was significantly reduced upon wIRA/VIS application at all evaluated irradiation intensities, suggesting contribution of host cell factors to the anti-chlamydial effect at the late stage of the chlamydial developmental cycle (Marti et al., 2015). Experiments in permanent cell lines (Marti et al., 2014, 2015) were performed in the presence of the host cell protein synthesis inhibitor cycloheximide, whereas in an additional follow-up study (Rahn et al., 2016) primary cell lines without cycloheximide supplementation were used.

The abovementioned preliminary results encouraged our further evaluation of wIRA/VIS as a potential non-chemical treatment method for trachoma, the most common cause of infectious blindness worldwide. Therefore, we employed an *in vitro* model for ocular chlamydial infections using the ocular *C. trachomatis* serovar B strain to infect human conjunctival epithelial cells (HCjE) and evaluated the effects of wIRA/VIS on non-infected ocular structures in two *ex vivo* eye models (Rahn et al., 2016). We demonstrated a significant wIRA/VIS-dependent reduction of chlamydial infectivity in HCjE. Unexpectedly, irradiation of HCjE prior to chlamydial infection was sufficient to inhibit chlamydial infectivity, suggesting the induction of a protective effect in wIRA/VIS-irradiated cells. Considering potentially harmful effects, wIRA/VIS irradiation did not reduce cell viability and post-treatment retinal damage was not observed. Additionally, vitreal temperature during wIRA/VIS irradiation did not markedly exceed physiological eye temperatures, suggesting that hyperthermia-related lesions are unlikely (Rahn et al., 2016). Therefore, wIRA/VIS has shown considerable promise as a non-chemical method for the treatment of ocular chlamydial infections, namely blinding trachoma.

Though preliminary studies indicate that thermal as well as non-thermal mechanisms are involved in the anti-chlamydial effect of wIRA/VIS (Marti et al., 2015), our understanding of the working mechanism of wIRA/VIS remains limited. In the first investigation into this working mechanism, Marti et al. (2014) detected increased cytokine and chemokine levels after wIRA/VIS irradiation of HeLa cells, an effect congruent with cytokine and chemokine release after *C. trachomatis* infection. This suggests a potential anti-chlamydial effect of wIRA/VIS based on immunological mimicry of chlamydial infections. In this study, we therefore aimed to more specifically evaluate the potential anti-chlamydial role of cytokines upon wIRA/VIS irradiation of *C. trachomatis*-infected HeLa cells.

## Materials and Methods

### Host Cells and Media

**FIGURE 1:**
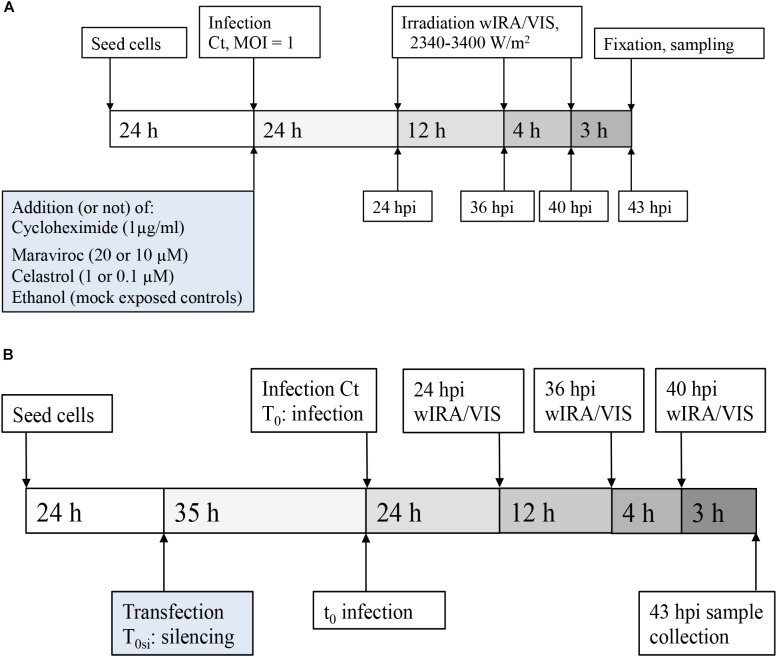
Experimental design. **(A)** Experimental setting for pharmaceutical treatment: HeLa cells were seeded and cultured for 24 h before infection with MOI = 1 of *C. trachomatis* Serovar E. wIRA/VIS irradiation was performed at 24, 36, and 40 hpi, for 30 min application time of irradiation, ranging between 2340 and 3400 W/m^2^. Cell densities were 5 × 10^4^ for pharmaceutical inhibition of cytokines and corresponding controls and 3 × 10^5^ for cycloheximide supplementation and 2 × 10^5^ for controls, respectively. After three additional hours of incubation (43 hpi), sampling for further analyses (including direct IFA, titration by sub-passage, ELISA of supernatants) was performed. **(B)** Experimental setting for gene silencing: After seeding and culture of HeLa cells (density 2.5 × 10^4^ cells/well), gene silencing (or negative control siRNA transfection) was performed, followed by an additional incubation time of 35 h before infection with *C. trachomatis* Serovar E at MOI = 1. Irradiation and sampling time points were the same as described in **(A)**.

HeLa cells (Homo sapiens cervix adenocarcinoma, CCL-2 ATCC) were cultured at 37°C with 5% CO_2_ in growth culture medium for cell propagation. HeLa growth medium consisted of Minimal Essential Medium with Earle’s salts (MEM; GIBCO, Life Technologies, Carlsbad, CA, United States) supplemented with 4 mM GlutaMAX-I (GIBCO), 1% MEM Non-Essential Amino Acids (MEM NEAA; 100x, GIBCO) and 10% fetal calf serum (FCS; BioConcept, Allschwil, Switzerland). Medium used for infections contained the same components as growth medium, but was not supplemented with fetal calf serum. Cells were seeded directly into wells or on round glass coverslips (13 mm diameter, Thermo Fisher Scientific, Waltham, MA, United States) in 24-well plates [Techno Plastic Products AG (TPP), Trasadingen, Switzerland]. Infection experiments were performed 24 h post-seeding or 59 h post-seeding (Figure 1).

### Chlamydial Strain

The genital strain *Chlamydia trachomatis* Serovar E (E/UW-5/CX kindly provided by Prof. R. V. Schoborg, Johnson City, TN, United States) was used for *in vitro* infection experiments. The isolate of the *C. trachomatis* strain was originally obtained from S. P. Wang and C.-C. Kuo (University of Washington, Seattle, WA, United States) and was propagated and harvested as described previously (Leonard et al., 2015). Briefly, chlamydia stocks were grown in HeLa cells (CCL-2, ATCC) at 37°C in HeLa growth medium supplemented with 1 μg/ml cycloheximide (CHX) in an atmosphere of 5% CO_2_ for 46 h, harvested and stored at -80°C in SPG medium (218 mM sucrose; Sigma-Aldrich, St. Louis, MO, United States), 3.76 mM KH_2_PO_4_ (Sigma-Aldrich), 7.1 mM K_2_HPO_4_ (Merck Eurolab AG, Dietlikon, Switzerland) and 5 mM GlutaMAX-100 (GIBCO). For infection, HeLa cells were seeded in 24-well plates and grown for 24 h at 37°C, 5% CO_2_. Infections were performed at a multiplicity of infection (MOI) of 1 with subsequent centrifugation at 1,000 ×*g* for 1 h at 25°C as described previously (Rahn et al., 2016).

### wIRA/VIS Irradiation

Cultures were irradiated three times at 24, 36, and 40 hpi with water-filtered infrared A combined with visible light (wIRA/VIS) for 30 min using a wIRA radiator (hydrosun 750, Hydrosun GmbH, Müllheim, Germany) at intensities ranging from 2340 to 3400 W/m^2^ (Figure 1). The resulting radiation spectrum ranges from 380 nm up to 1,400 nm. The irradiation procedure, cooling system and non-irradiated controls were the same as previously described (Jung et al., 2010; Marti et al., 2014). Briefly, 24-well plates were placed into a thermostat-controlled water bath (SC100, Thermo Fisher Scientific) set to 37°C. Non-irradiated controls were placed on the same plates with suitable distance from irradiated conditions to avoid any irradiation influence. Irradiation was guided through optical fibers, reaching from the wIRA radiator (emission source) to the surface of the appropriate wells (irradiator point).

### ELISA for IL-6, IL-8, and RANTES/CCL5

At 43 hpi, cell culture supernatants were collected, filtered through 0.1 μm syringe filters (Whatman Anotop, Sigma-Aldrich) and stored at -80°C until further processing. Cytokine levels were determined using human ELISA kits for IL-6 (ID: KHC0061), IL-8 (ID: EH2IL8), and RANTES (ID: EHRNTS) according to manufacturer’s instructions [Invitrogen^TM^ (IL-6, RANTES) Carlsbad, CA, United States, Thermo Fisher Scientific (IL-8)]. Absorbance endpoint plate reading was performed on an Epoch 2 Microplate Spectrophotometer (BioTek^®^, Winooski, VT, United States) at 450 nm wavelength, blank corrected and evaluated by the four-parameter logistic ELISA curve fitting provided by elisaanalysis.com. Cytokine concentrations were calculated in Microsoft Excel (Microsoft, Redmont, WA, United States) as average ± SD and expressed as percentage of untreated controls. Since RANTES (also called CCL5) represents a chemokine in the group of cytokines, it will be further referred to as cytokine.

### Study Design

#### Infection Experiments

The experiments were organized in four treatment groups: (a) mock-infected and non-irradiated, (b) mock-infected and irradiated, (c) *C. trachomatis*-infected and non-irradiated, and (d) *C. trachomatis*-infected and irradiated. Centrifugation-assisted infection of monolayers was performed as previously described (Rahn et al., 2016), with replacement of the inoculum with cycloheximide (CHX)-containing or CHX-free medium after infection (time point 0) depending on the experimental setting, and the infected cells were incubated for 40 h, during which time three applications of irradiation were administered (Figure 1). Post-irradiation, an incubation for three additional hours was applied and at 43 hpi, monolayers of all four groups were either fixed with methanol for immunofluorescence assay (IFA, on glass slides) or collected for titration by sub-passage as previously described (Marti et al., 2014). IFA was performed to label chlamydial LPS as previously described (Rahn et al., 2016) and chlamydial inclusion morphology was assessed. To determine mean inclusion size, 50 randomly selected inclusions were examined per condition and area in μm^2^ was calculated using BonTec measuring and archiving software (BonTec, Bonn, Germany; Leonard et al., 2015). Number of inclusion forming units (IFU) per mL was evaluated by sub-passage to determine infectious chlamydial particles as described in detail elsewhere (Rahn et al., 2016). IFU/mL was calculated and expressed as percentage of the corresponding control. Unless stated otherwise, experimental values were determined from duplicates, three independent experiments were performed, and data was expressed as the average ± standard deviation (SD) of three experimental values. Measurements were tested for normal distribution by *Shapiro–Wilk test* in R (R Core Team, 2016) and statistical analysis was performed using *Wilcoxon Rank Sum test* with *p*-values <0.05, <0.01 or <0.001 for significant differences.

#### Cycloheximide and Irradiation Experiments (Figure 1A)

HeLa cells were seeded at a density of 2 × 10^5^ cells/well for conditions without CHX or 3 × 10^5^ cells/well for CHX-exposed conditions and incubated for 24 h before infection with *C. trachomatis* Serovar E at a MOI of 1. After centrifugation, infection media were replaced by cycloheximide-containing (1 μg/ml, CHX) or cycloheximide-free (CHX-free) incubation medium and further processed as described above.

### Silencing RNA (siRNA) for IL-6, IL-8, and RANTES: Transfection Procedure, Quantitative Real-Time PCR, and Irradiation Experiments (Figure 1B)

#### Transfection Procedure

Transfection of HeLa cells was performed according to Ambion manufacturer’s guidelines (Life technologies) in 24-well culture plates using 6.25 nM siRNA and 2 μl Lipofectamine^®^ RNAiMAX reagent for each siRNA (Thermo Fisher Scientific) under normoxic conditions in OptiMEM I reduced serum medium (Thermo Fisher Scientific).

A mixture of two different silencer select siRNA for each cytokine (IL-6: ID: s7311, s7313; IL-8: ID: s7328, s7327; RANTES: ID: s12575, s12577) with a final concentration of 12.5 nM siRNA and 4 μl of Lipofectamine^®^ RNAiMAX reagent and one silencer^®^ select negative control (#1 siRNA) with a final concentration of 6.25 nM siRNA and 2 μl Lipofectamine^®^ RNAiMAX reagent and dissolved in OptiMEM to a total volume of 400 μl per well was used. At the time of transfection, cell layers were reaching a confluency of 20–30%. After an incubation time of 5 h, transfection reagents were replaced by usual HeLa growth medium without CHX for the further incubation of cells.

Knockdown efficacy was confirmed by quantitative polymerase chain reaction (qPCR) at 24, 48, 72, and 90 h post-transfection in a preliminary experiment with two silenced wells (duplicates) per time point and cytokine (see the Section “Quantitative Real-Time PCR”). Transfection time point for irradiation experiments was 24 h post-seeding and 35 h prior to infection.

#### Quantitative Real-Time PCR

Total RNA was isolated using the RNeasy mini kit (ID: 74104, Qiagen, Venlo, Netherlands) and RNA content was measured by a NanoDrop Microvolume Spectrophotometer and Fluorometer (NanoDrop Technologies, LLC, Wilmington, DE, United States). 150 ng of extracted RNA was reverse transcribed into cDNA using the high-capacity cDNA reverse transcription kit (Applied Biosystems, Foster City, CA, United States) according to manufacturer’s guidelines (concentration of 15 ng/μl) on a Biometra Trio Thermocycler (Analytik Jena, Jena, Germany).

PCR amplification was performed using the TaqMan^TM^ Gene Expression Assays for IL-6 (Hs00174131_m1), IL-8 (CXCL8, Hs00174103_m1) and RANTES (Hs00982282_m1) and the TaqMan^®^ Fast Universal PCR Master Mix [2x] (Applied Biosystems) on a 7500- Fast ABI Thermocycler (Applied Biosystems). Cycle protocol was set according to manufacturer’s instructions.

Silenced samples were run in triplicates (RANTES) or quadruplicates (IL-6, IL-8). Molecular-biology-grade water (Thermo Fisher Scientific) served as no template controls (NTC), which were run in duplicate, as were mismatch-silenced controls. Relative quantification of IL-6, IL-8, and RANTES mRNA expression was determined relative to the endogenous control human Actin Beta [Human ACTB endogenous Control 4310881E (probe VIC, quencher TAMRA), Thermo Fisher Scientific] using the 2^-ΔΔCT^ method. Data was calculated relative to the mismatch-silenced control mRNA levels using Microsoft Excel (Microsoft).

#### Irradiation Experiments With Transfected Cells

HeLa cells were seeded at a density of 2.5 × 10^4^ cells/well and incubated for 24 h before transfection. Transfection was performed as stated in “transfection procedure” and cells were infected (or mock-infected) 35 h post-transfection. Non-transfected cells (data not shown) and mismatch transfected cells served as controls. After centrifugation, infection media were replaced by CHX-free incubation medium and irradiation was carried out as described in the Sections “wIRA/VIS Irradiation” and “Infection Experiments.”

### Pharmaceutical Inhibition of Cytokines: Cell Viability Assays, Inhibitor Concentration Curves, and Irradiation Experiments (Figure 1A)

Cell densities for all experiments with pharmaceutical inhibition of cytokines were 5 × 10^4^ cells/well. Ethanol (100%) was filtered through a 0.22 μm PES-membrane syringe filter (Techno Plastic Products AG [TPP]) and used as solvent control or to dilute pharmaceutic reagents. Based on the results of the cell viability assays (see below), ethanol concentrations over 0.5% were not used for further experiments. Celastrol 10 mg c0869 (Sigma-Aldrich), an inhibitor of IL-6 and IL-8, was dissolved in 1 ml of 100% ethanol to a stock concentration of 10 mg/ml. Maraviroc 10 mg 3756 (Tocris, Bristol, United Kingdom), a selective CCR5 receptor antagonist (and therefore an inhibitor of RANTES/CCL5), was dissolved in 1 ml of 100% ethanol to a stock concentration of 10 mg/ml. Stocks were re-filtered through 0.22 μm PES-membrane syringe filters (TPP) and then stored light-protected at -20°C until further use.

#### Cell Viability Assays

Cell viability assays were run in triplicates: Cells were incubated for 24 h before adding a range of concentrations of ethanol, Maraviroc and Celastrol, depending on the experimental setting. Cell viability under ethanol incubation was tested by mixing 100% ethanol in a 1:1 ratio with sterile phosphate-buffered saline (PBS – GIBCO) and tested in HeLa growth medium at concentrations of 10%, 5%, 1%, 0.5%, and 0.1%. The resulting final concentrations of ethanol were 5%, 2.5%, 0.5%, 0.25%, and 0.05%. Maraviroc was tested at concentrations of 20 μM, 10 μM, 1 μM, and 0.1 μM and 0.103% ethanol in growth medium served as the Maraviroc solvent control. Celastrol was tested at concentrations of 25 μM, 2 μM, 1 μM and 0.1 μM and 0.113% ethanol in growth medium served as the Celastrol solvent control. 10% Alamar blue dye (Invitrogen^TM^) was added to cell cultures at 12, 24, 36, and 45 h (ethanol) or 48 h (Maraviroc/Celastrol) after incubation. After 3 h of incubation at 37°C, 2 × 200 μl, Alamar blue dye/culture medium was transferred into 96 well plates (generating two replicates from each well) and fluorescence was monitored using a Synergy HT Reader 270230 (BioTek^®^) at 530-nm excitation and 590-nm emission wavelength. Raw data were analyzed as mean ± SD in Microsoft Excel (Microsoft) and expressed as percentage of controls. If not stated differently, heat denatured cells served as positive controls.

#### Inhibitor Concentration Curves

To determine potential effects of the solvent (ethanol) and pharmaceutic inhibitors on *C. trachomatis*, infections were performed with supplemented media at the same concentrations as in the cell viability assays in triplicates, using 0.113% (Celastrol) or 0.103% (Maraviroc) ethanol-containing medium as corresponding mock-exposed controls.

Briefly, *C. trachomatis* stocks were diluted in infection media supplemented with the appropriate concentrations of ethanol, Celastrol or Maraviroc, to MOI of 1 and centrifuged for 1 h at 25°C and 1,000 ×*g* (Rahn et al., 2016). After centrifugation, infection media were replaced by incubation medium with corresponding ethanol, Celastrol or Maraviroc concentrations. Cells were incubated for an additional 43 h, then fixed with methanol as described previously (Marti et al., 2014). Inclusion size and morphology were analyzed as described above. Inclusion numbers were analyzed by counting inclusion numbers in 30 randomly selected view fields at 200-fold magnification. Mean ± SD for each concentration were calculated in Microsoft Excel (Microsoft) and expressed as percentage of untreated control (data not shown) or mock-exposed controls.

#### Irradiation Experiments

Celastrol concentrations of 1 μM and 0.1 μM, and Maraviroc concentrations of 20 μM and 10 μM were chosen for irradiation experiments, based on the results of the cell viability assays and inhibitor concentration curves. Corresponding volumes of 100% ethanol were used as mock-exposed controls reaching final ethanol concentrations of 0.045% (1 μM and 0.1 μM Celastrol), 0.103% (20 μM Maraviroc) and 0.0515% (10 μM Maraviroc). Infection media and incubation media (CHX-free) were supplemented with the inhibitors or 100% ethanol and the same infection-, irradiation- and sampling steps as in previous irradiation experiments were performed (see above).

## Results

### wIRA/VIS Reduces Chlamydial Infectivity Independent of Cycloheximide (CHX) and Reduces Chlamydial Inclusion Size (Figure 2)

**FIGURE 2:**
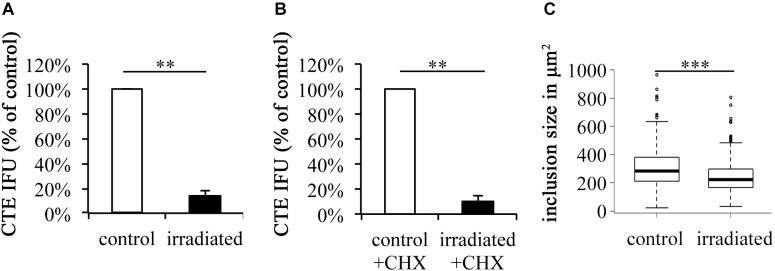
wIRA/VIS reduces chlamydial infectivity independent of cycloheximide (CHX) and reduces chlamydial inclusion size. According to Figure 1, irradiation experiments were performed. Experiments were run either without **(A)** or with **(B)** cycloheximide (CHX) supplementation following chlamydial infection. Chlamydial infectivity upon titration by sub-passage was determined as IFU/ml and is presented in the black bars as percentage of non-irradiated controls (empty bars). **(C)** Demonstrates the reduction of chlamydial inclusion size upon wIRA/VIS irradiation (*n* = 9). Significance levels are marked with asterisks: ^∗^*p* < 0.05, ^∗∗^*p* < 0.01, and ^∗∗∗^*p* < 0.001.

To rule out potential effects of CHX, an eukaryote protein synthesis inhibitor, on the irradiation efficacy of wIRA/VIS, CHX-containing and CHX-free conditions were compared. Chlamydial infectivity after irradiation was significantly reduced, independent of CHX-addition, resulting in remaining chlamydial infectivity of 10.14% (±0.81%) in CHX-containing conditions (*p*-value < 0.01) and 13.94% (±4.70%) in CHX-free conditions (*p*-value < 0.01; Figures 2A,B). The absolute chlamydial loads were significantly higher (*p*-value < 0.01) in the CHX-supplemented conditions compared to the CHX-free ones. CHX-containing controls reached 6.56 × 10^7^ ± 1.60 × 10^7^ IFU/ml whereas the CHX-free conditions with 1.18 × 10^6^ ± 2.61 × 10^5^ IFU/ml contained about 50 times less chlamydia. wIRA/VIS irradiated conditions with CHX (6.63 × 10^6^ ± 1.43 × 10^6^ IFU/ml) and without CHX (1.62 × 10^5^ ± 4.67 × 10^4^ IFU/ml) displayed the same significance level (*p*-value < 0.01). Furthermore, irradiated inclusions were visibly smaller than those in the corresponding controls, which was further analyzed by comparing inclusion sizes of untreated controls with irradiated samples (*n* = 9). Mean inclusion sizes of non-irradiated inclusions (305.47 μm^2^, ± 135.35 μm^2^) differed significantly from those of irradiated inclusions (238.59 μm^2^, ±111.00 μm^2^) as visible in Figure 2C (CHX-free, *p*-value < 0.001).

### Cytokine Secretion (IL-6, IL-8, RANTES) Upon wIRA/VIS Treatment Is Independent of CHX (Figure 3)

**FIGURE 3:**
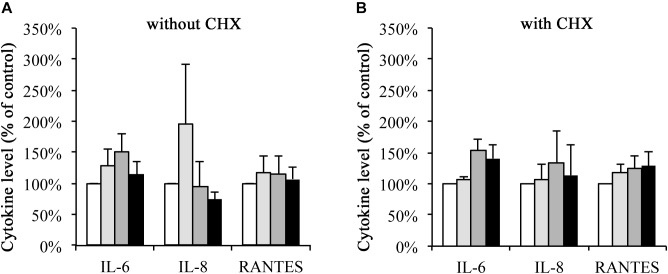
Cytokine secretion of IL-6, IL-8, and RANTES upon wIRA/VIS treatment is independent of CHX. After seeding, infection and irradiation without **(A)** or with **(B)** cycloheximide (CHX) supplementation, supernatants were collected for IL-6, IL-8, and RANTES ELISA analysis at 43 hpi. IL-6, IL-8, and RANTES levels are expressed according to the experimental groups as percentage of non-irradiated controls. Treatment groups include mock-infected and non-irradiated conditions (controls, empty bars), mock-infected and irradiated (bright gray bars), *C. trachomatis*-infected and non-irradiated (dark gray bars) and *C. trachomatis*-infected and irradiated (black bars) conditions.

HeLa cell secretion of IL-6, IL-8, and RANTES into the culture media was analyzed upon infection, irradiation, or the combination of both, and compared to the mock-infected non-irradiated control. In the absence of CHX (Figure 3A), IL-6 levels of 128.50% (±26.91%) upon irradiation, 149.96% (±30.98%) upon infection and 114.66% (±19.86%) upon the combination of both were observed. Upon irradiation, IL-8 levels increased to 195.62% (±97.25%) but were decreased upon *C. trachomatis* infection (95.46% ± 40.63%) and upon infection and irradiation (74.35% ± 10.66%). RANTES secretion was increased in all three experimental conditions (118.27% ± 25.82% upon irradiation, 115.24% ± 28.53% upon infection, 107.22% ± 20.16% upon the combination of both).

In the presence of CHX (Figure 3B), IL-6 secretion was increased upon irradiation (106.24% ± 5.86%), infection (154.20%, ± 18.28%) or the combination of both (140.70% ± 22.14%). IL-8 levels showed increases to 107.32% (±23.19%) upon irradiation, 134.58% (±50.57%) upon infection and 113.74% (±48.12%) upon the combination of both. And, finally, RANTES levels increased to 118.66% (±12.83%) upon irradiation alone, 125.47% (±20.23%) upon infection alone and 128.07% (±24.20%) after irradiation and infection.

### Gene Silencing of IL-6, IL-8, and RANTES Downregulates mRNA Levels of All Targets Over a Time Period of 90 h (Supplementary Figure S1)

Decreases in mRNA levels were confirmed over a maximum time period of 90 h post-transfection to ensure that reduced mRNA levels of IL-6, IL-8, and RANTES were reached over the duration of the irradiation experiments. Mismatch negative silencer controls were evaluated at the same time points and set to 100% gene expression. Gene expression levels of IL-6 began decreasing by 24 h (61.08% ± 4.55%) and dropped to 32.50% of control (±17.28%) after 48 h, 24.96% (±6.39%) after 72 h and 27.80% (±0.30%) after 90 h. mRNA levels of IL-8 dropped to 10.83% (±5.85%) within 24 h and reached 37.89% (±4.01%) of control after 48 h, 43.44% (±2.35%) after 72 h and 33.71% (±3.67%) after 90 h. RANTES mRNA dropped to 24.87% (±12.46%) at 24 h, 8.67% (±1.35%) at 48 h, 7.71% (±1.43%) at 72 h and 23.90% (±10.89%) at 90 h post-transfection.

### The Reduction of Chlamydial Infectivity by wIRA/VIS Is Independent of IL-6, IL-8, or RANTES Gene Silencing (Figure 4)

**FIGURE 4:**
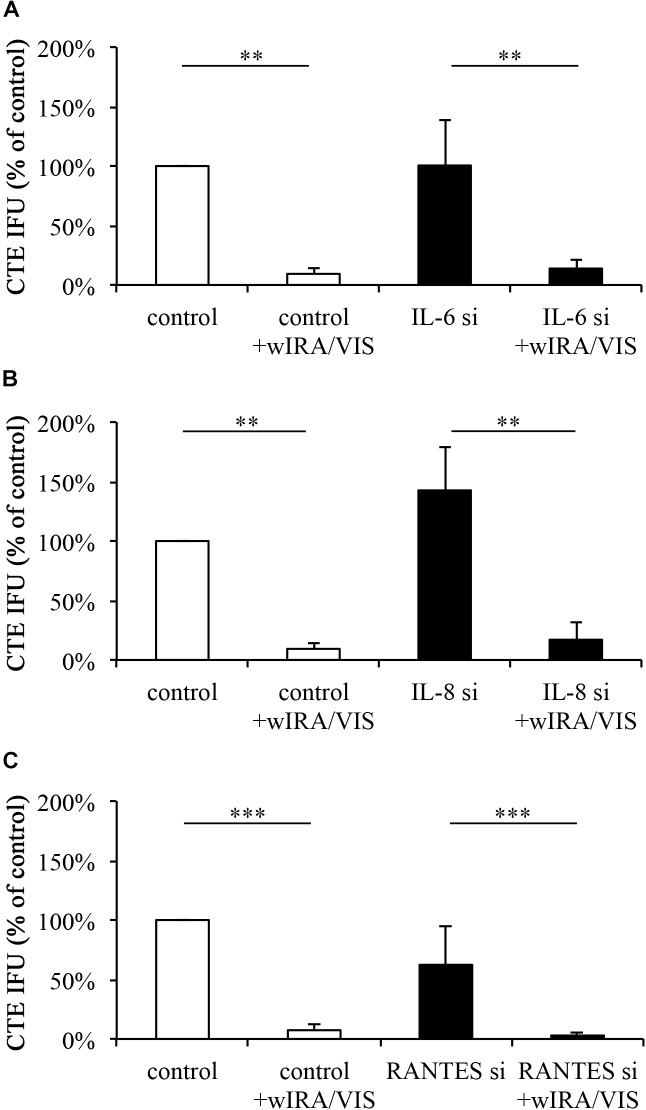
Gene silencing of IL-6, IL-8, and RANTES does not influence the anti-chlamydial effect of wIRA/VIS. After seeding, gene silencing for IL-6 **(A)**, IL-8 **(B)**, and RANTES **(C)**, infection with *C. trachomatis* serovar E and irradiation, monolayers were sampled for titration by sub-passage to evaluate chlamydial infectivity. Chlamydial infectivity, as IFU/ml, was determined and expressed as percentage of non-irradiated control samples. Chlamydial infectivity was significantly decreased in all irradiated conditions, independent of gene silencing. Figures show results of three independent experiments for IL-6 and IL-8 silencing and five experiments for RANTES gene silencing. Significance levels are marked with asterisks: ^∗^*p* < 0.05, ^∗∗^*p* < 0.01, and ^∗∗∗^*p* < 0.001.

Mismatch controls and target silenced cells were infected and irradiated as described above. Using sub-passage titer assays, chlamydial infectivity was calculated and expressed as percent of non-irradiated, mismatch silenced controls. Infection of IL-6 silenced cells resulted in chlamydial infectivity of 100.48% (±38.81%; *p*-value > 0.05) of the control. wIRA/VIS irradiation at 24, 36, and 40 hpi resulted in a significant reduction of the chlamydial infectivity to 9.36% (±5.44%) for controls and 13.85% (±7.91%) in IL-6 gene silenced cells (*p*-values < 0.01; Figure 4A).

Infection of IL-8 silenced cells resulted in increased, but not significant (*p* > 0.05) chlamydial loads of 142.61% (±36.79%). wIRA/VIS treatment of infected cells reduced chlamydial infectivity significantly to 9.36% (±5.44%) in controls and 17.04% (±15.04%) in IL-8 silenced cells (*p* < 0.01; Figure 4B).

Infection of RANTES silenced cells resulted in chlamydial loads of 62.36% (±32.71%), which was not statistically significant compared to controls. Irradiation reduced chlamydial infectivity significantly (*p* < 0.001) to 7.37% (±4.36%) in controls and 2.91% (±2.91%) in RANTES silenced conditions (*n* = 5, Figure 4C).

### Increasing Ethanol and Celastrol Concentrations Decrease Cell Viability While Maraviroc Concentrations up to 20 μM Have No Effect on Cell Viability (Supplementary Figure S2)

Ethanol was employed as the diluent for Celastrol and Maraviroc in this study. Five ethanol concentrations (0.05%, 0.25%, 0.5%, 2.5% and 5.0%) were analyzed for potential impact on host cell viability at 12, 24, 36, and 45 h post-incubation, as measured by Alamar blue assay. At all ethanol concentrations, decreased cell viability, compared to the control, was present at 24 h post-incubation. However, cell viability at 36 and 45 h post-incubation was increased or slightly decreased at ethanol concentrations up to 0.5%, compared to controls, while concentrations of 2.5% and 5% reduced cell viability at all evaluated time points (Supplementary Figure S2A).

Ethanol concentrations in Celastrol and Maraviroc dilutions reached 0.113% and 0.103%, respectively, and were reflected in the diluent controls for each inhibitor. Celastrol reduced cell viability at concentrations of 2 μM and 25 μM (Supplementary Figure S2B). Measured decrease in cell viability by Alamar blue assay was accompanied by the presence of increased numbers of rounded, detaching and floating cells and reduced cell confluency (data not shown). Therefore, Celastrol concentrations of 1 μM and 0.1 μM were chosen for irradiation experiments.

Maraviroc, even at the high concentration of 20 μM and the longest incubation time of 48 h post-incubation, d id not induce any change in cellular morphology (data not shown) or cell viability, thus, concentrations of 10 μM and 20 μM Maraviroc were chosen for irradiation experiments (Supplementary Figure S2C).

### Celastrol Reduces *C. trachomatis* Inclusion Number and Size, Whereas Maraviroc Only Reduces Inclusion Number (Figure 5)

**FIGURE 5:**
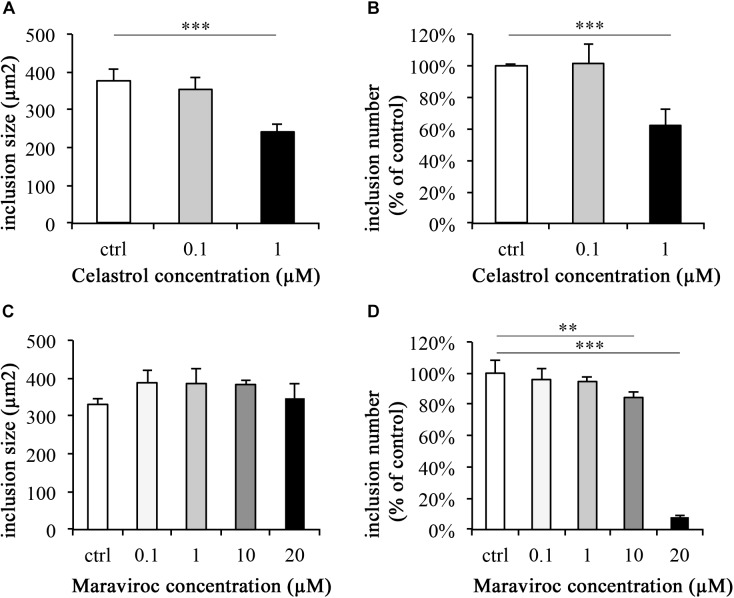
Celastrol significantly reduces *C. trachomatis* inclusion number and size, whereas Maraviroc reduces the inclusion number. *C. trachomatis* infection and supplementation with Celastrol **(A,B)** and Maraviroc **(C,D)** were performed in parallel. After 43 h of incubation, monolayers were fixed with methanol and immunofluorescence stained. Inclusion sizes **(A,C)** and numbers **(B,D)** were analyzed by either measurement of inclusions or counting of inclusion numbers per fields (200× magnification) relative to controls (ctrl). Significance levels are marked with asterisks: ^∗^*p* < 0.05, ^∗∗^*p* < 0.01, and ^∗∗∗^*p* < 0.001.

As determined by IFA analysis of inclusion morphology, none of the evaluated ethanol, Celastrol or Maraviroc concentrations induced signs of persistence such as AB formation (data not shown). At 43 hpi, inclusion sizes in controls were 376.05 μm^2^ (±30.93 μm^2^) and 0.1 μM Celastrol incubated inclusions were of similar size, 353.86 μm^2^ (±30.61 μm^2^). A significant reduction in inclusion size (241.79 μm^2^, ± 20.00 μm^2^) was observed at a concentration of 1 μM Celastrol (*p* < 0.001, Figure 5A). Inclusion numbers were significantly reduced at 1 μM Celastrol incubation (62.10% ± 9.96%, *p* < 0.001), whereas no significant difference was seen at a Celastrol concentration of 0.1 μM (101.38% ± 12.56%; Figure 5B).

None of the evaluated concentrations of Maraviroc led to a significant reduction of inclusion size compared to the control (Figure 5C). Inclusion numbers were significantly decreased at 10 μM Maraviroc treatment to 84.45% (±3.05%, *p*-value < 0.01) and at 20 μM (7.97% [±0.69%], *p*-value < 0.001, Figure 5D). No significant differences from the control were observed at Maraviroc concentrations of 0.1 μM or 1 μM.

### The Reduction of Chlamydial Infectivity by wIRA/VIS Is Independent of Pharmaceutical Cytokine Inhibition by Celastrol or Maraviroc (Figure 6)

**FIGURE 6:**
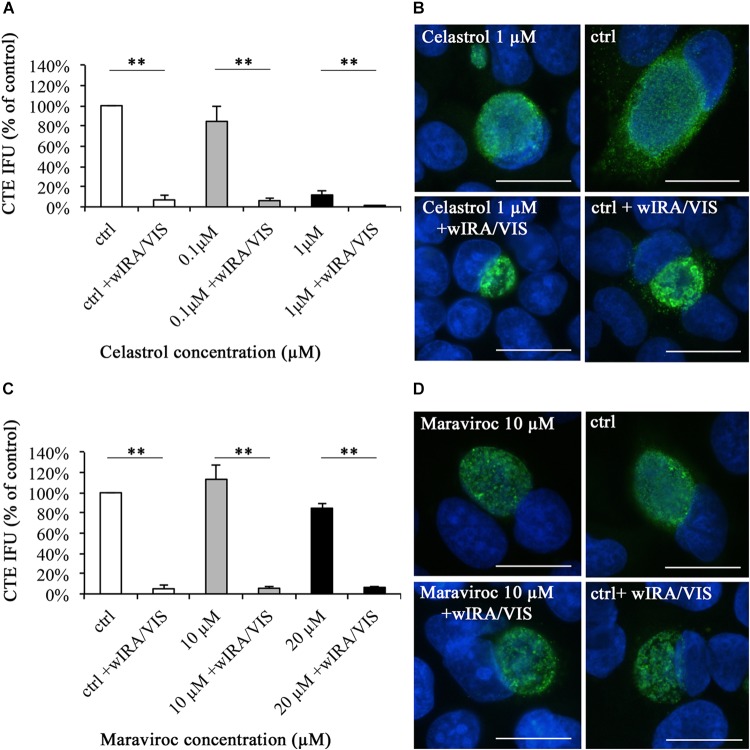
The pharmaceutical inhibition of IL-6, IL-8, or RANTES does not abolish the anti-chlamydial effect of wIRA/VIS on *C. trachomatis.* After seeding, infection, Celastrol **(A,B)** or Maraviroc **(C,D)** supplementation and irradiation, monolayers were either fixed with methanol for direct IFA analysis **(B,D)** or sampled for titration by sub-passage to determine chlamydial infectivity **(A,C)**. Chlamydial infectivity was calculated as IFU/ml and expressed as percentage of the non-irradiated control (ctrl). Bars represent results of three independent experiments. For Maraviroc **(C)**, the two investigated concentrations were tested in separate experimental settings and results were summarized into one figure, resulting in *n* = 6 for the control (ctrl). Significance levels are marked with asterisks: ^∗∗^*p*-values < 0.01. To assess potential morphological changes under pharmaceutical inhibition, direct IFA of monolayers was performed and analyzed with regards to inclusion morphology and size. Celastrol 1 μM alone led to significantly smaller inclusions compared to controls (ctrl) **(B)**, whereas Maraviroc did not alter inclusion morphology **(D)**.

Infective chlamydial loads were determined by sub-passage titer assays and were calculated and expressed as percentage of the control. Utilizing inhibitor concentrations with minimal negative impact on cell viability [as described in the Sections “Inhibitor Concentration Curves” and “Increasing Ethanol and Celastrol Concentrations Decrease Cell Viability While Maraviroc Concentrations up to 20 μM Have no Effect on Cell Viability (Supplementary Figure S2)”] and associated with the observed inhibitor-dependent decreases in inclusion numbers discussed above, Celastrol incubation alone reduced chlamydial loads. Samples incubated with 0.1 μM Celastrol showed reduced infectivity of 84.47% (±14.51%) of the control, whereas 1 μM Celastrol resulted in a significant reduction (*p* < 0.01) of chlamydial infectivity to 11.77% (±4.57%) of the control. Irradiation of *Chlamydia*-infected cells with wIRA/VIS further decreased chlamydial infectivity to 6.89% (±4.32%) for the controls, 6.15% (±2.28%) at 0.1 μM Celastrol, and to 0.77% (±0.39%) at a concentration of 1 μM Celastrol. Statistical analysis showed significant differences for the reduction of chlamydial infectivity upon irradiation in all three tested conditions, with *p*-values of < 0.01 (Figure 6A).

*C. trachomatis*-infected inclusions incubated with 1 μM Celastrol were smaller than control inclusions (Figure 6B) and irradiation reduced inclusion sizes independent of Celastrol treatment (data not shown) as observed for control samples (Figure 2C).

Maraviroc treatment resulted in chlamydial infectivity of 113.24% (±7.68%) at 10 μM and 84.70% (±31.18%) at 20 μM concentrations, respectively, compared to the control. Irradiation with wIRA/VIS reduced chlamydial infectivity significantly in all evaluated conditions (*p* < 0.01): Remaining infectivity after irradiation was 5.06% (±2.24%) in controls (*n* = 6 for controls), 5.57% (±4.53%) in 10 μM and 6.46% (±5.19%) in 20 μM Maraviroc conditions (Figure 6C). Maraviroc incubation alone did not influence chlamydial inclusion morphology (Figure 6D).

## Discussion

Triple irradiation with wIRA/VIS during the course of *C. trachomatis* infection significantly reduces chlamydial infectivity in HeLa cells, which is in accordance with previous studies (Marti et al., 2014, 2015; Rahn et al., 2016). CHX, a host protein synthesis inhibitor, is known to promote chlamydial infection (Ripa, 1977; Wyrick, 2010), which was also confirmed in our study [see the Section “wIRA/VIS Reduces Chlamydial Infectivity Independent of Cycloheximide (CHX) and Reduces Chlamydial Inclusion Size (Figure 2)”]. Comparison of CHX-containing and CHX-free irradiated conditions revealed similar effects of wIRA/VIS treatment compared to the corresponding controls, indicating that wIRA/VIS irradiation is independent of CHX in our *in vitro* model (Figure 2).

The secretion of pro-inflammatory cytokines in response to wIRA/VIS irradiation alone was independent of CHX incubation (Figure 3): in the presence or absence of CHX, wIRA/VIS-dependent increase in secretion of IL-6, IL-8, or RANTES was, although not statistically significant, consistently observed. This was in accordance with previous results of cytokine regulation under wIRA/VIS irradiation (Marti et al., 2014). Dessus-Babus et al. (2000) and Leonard et al. (2017) reported increased IL-6 secretion after chlamydial infection under CHX-influence, which we could confirm after comparing CHX-free and CHX-incubated conditions by *t*-test (data not shown). IL-8 secretion, however, was previously shown to decrease after CHX-treatment of *Chlamydia*-infected host cells (Dessus-Babus et al., 2000), which was not confirmed in our study.

In this study, we did not report significantly increased levels of IL-6, IL-8, or RANTES upon chlamydial infection (alone or in combination with wIRA/VIS irradiation; Figure 3). The induction of IL-6 after *C. trachomatis* infection (in CHX-free conditions) has been observed by other authors (Rasmussen et al., 1997; Dessus-Babus et al., 2000; Gervassi et al., 2004; Cheng et al., 2008; Buckner et al., 2013). Similar results were obtained in multiple studies regarding IL-8 secretion (Rasmussen et al., 1997; Gervassi et al., 2004; Buchholz and Stephens, 2006; Cheng et al., 2008; Buckner et al., 2013). Dessus-Babus et al. (2000) also studied HeLa cells infected with *C. trachomatis* Serovar E and found increased IL-8 levels in the absence of CHX only after 48 hpi (not at earlier time points), but polarized cells were used in contrast to the non-polarized cells in our study. Even though Buckner et al. (2013) observed increases of IL-6 and IL-8, they did not reproduce the magnitude of up-regulation observed by Rasmussen et al. (1997).

Increased RANTES levels after *C. trachomatis* infection was detected in investigations of the genital tract of mice infected with *C. trachomatis* (Maxion and Kelly, 2002). In contrast, Buckner et al. (2013) found decreased levels of RANTES in primary endocervical epithelial cells after infection with *C. trachomatis* Serovar D at 72 hpi.

Regardless of CHX-dependent effects on cytokine responses to chlamydial infection, the increased cytokine levels after wIRA/VIS irradiation, even though not significant, were a constant finding for all investigated cytokines, and resembled the detected cytokine release pattern reported by Marti et al. (2014). Considering that chlamydiae are a known trigger for cytokine secretion, their amount may also have a direct influence on cytokine secretion levels (Maxion and Kelly, 2002). Even though all infection steps were performed with MOI = 1 in our experiments, wIRA/VIS irradiation led to reduced chlamydial infectivity in the samples, which potentially influences the amount of secreted cytokines (Maxion and Kelly, 2002). Therefore, a theoretical correction of cytokine levels according to chlamydial infectivity was performed and resulted in significantly increased levels for all investigated cytokines under infection and irradiation treatment (data not shown). This supported our hypothesis that pro-inflammatory cytokines might play a role in wIRA/VIS-dependent anti-chlamydial effects.

Targeted cytokine suppression in HeLa cells was achieved by respective gene silencing (Supplementary Figure S1). The introduction of double-stranded RNAs into mammalian cells can lead to type I interferon (IFN) reactions involving IFN-α and -β (Matsumoto et al., 2004) and is known to evoke non-specific toxic effects in host cells (e.g., resulting in cell death or general shut-down of host cell protein synthesis) (Wadhwa et al., 2004; Seok et al., 2018), even though the used siRNAs in our study are considered to induce minimal side effects (manufacturer’s manuals). Interferons such as IFN-γ and host cell factors such as amino acid starvation or iron deprivation are known inducers of the so-called chlamydial stress response (Wyrick, 2010; Schoborg, 2011). In view of this, we qualitatively evaluated chlamydial inclusions upon chlamydial infection of target and mismatch control silenced HeLa cells, with regards to potential inclusion size reduction such as seen under DAMP influences (Leonard et al., 2015), or regarding morphological features indicative of AB formation but neither effect was observed.

Cytokine gene silencing did not have a significant effect on chlamydial infectivity [see the Section “The Reduction of Chlamydial Infectivity by wIRA/VIS Is Independent of IL-6, IL-8, or RANTES Gene Silencing (Figure 4)”] or cell viability (compared to mismatch silenced conditions). However, wIRA/VIS irradiation following gene silencing led to a reduction of chlamydial infectivity similar to that observed in irradiated mismatch silenced and non-transfected controls (Figure 4). This indicates that the wIRA/VIS anti-chlamydial effect is not abolished when mRNA transcription of pro-inflammatory cytokines such as IL-6, IL-8, and RANTES is suppressed.

Next, we investigated the effect of pharmaceutical cytokine suppression on the wIRA/VIS-dependent anti-chlamydial effect by using commercially available cytokine inhibitors. Celastrol is a natural substance derived from Thunder of God Vine (*Tripterygium wilfordii*) and used in traditional Chinese medicine (Kannaiyan et al., 2011; Venkatesha et al., 2016). Celastrol has been demonstrated to promote broad anti-inflammatory and anti-cancer effects (Kannaiyan et al., 2011; Venkatesha et al., 2016). In our study, we used Celastrol as an inhibitor for IL-6 and IL-8 secretion. In preliminary experiments, potential negative effects of Celastrol on HeLa cells were evaluated by performing cell viability assays (Supplementary Figure S2). Celastrol concentrations of 2 μM or higher reduced cell viability, therefore, 1 μM and 0.1 μM were chosen for subsequent irradiation experiments. Chiang et al. (2014) observed significantly reduced cell proliferation in prostate cancer cell lines for 0.1 μM concentration of Celastrol and were able to explain this finding by cell cycle inhibition and induced apoptosis. Shrivastava et al. (2015) observed similar findings in breast cancer cell lines at Celastrol concentrations ranging from 0.5 to 25 μM. Celastrol concentrations of 1 μM and 0.1 μM are considered effective for IL-6 and IL-8 inhibition according to one supplier’s manuals (Santa Cruz, recommended concentrations for Celastrol as cytokine inhibitor: IC_50_ for IL-6 inhibition = 80 nM, IC_50_ for IL-8 210 nM^+^).

First, we performed concentration curve analyses for Celastrol to gain insight into the effect of this inhibitor on chlamydial inclusion morphology, size and number (Figure 5). Morphology features indicative of persistence were not observed, but inclusion size and numbers were significantly reduced at 1 μM Celastrol concentration, compared to the control, whereas 0.1 μM concentration did not significantly change inclusion size or numbers. The addition of Celastrol decreased chlamydial infectivity, as assessed by sub-passage. This contrasts the results of cytokine inhibition by gene silencing, where inhibition of IL-6 or IL-8 gene silencing did not significantly influence chlamydial infectivity. To our knowledge, no studies including Celastrol application and *C. trachomatis* have been performed until now. Infectivity upon wIRA/VIS irradiation was invariably reduced in all evaluated groups. In conclusion, the anti-chlamydial effect of wIRA/VIS is independent of IL-6, IL-8 and pharmaceutical treatment with Celastrol (Figure 6).

Maraviroc is a competitive CCR5 inhibitor approved for treating HIV/AIDS patients (Xu et al., 2014; Vangelista and Vento, 2018). CCR5 receptors are expressed on many inflammatory cells (e.g., T-cells, macrophages or dendritic cells) and serve as an important entry and binding site for the human immunodeficiency virus (Oliveira et al., 2014; Xu et al., 2014). HeLa cells *in vitro* and cervical cancer samples *ex vivo* were demonstrated to express CCR5 (Sales et al., 2014; Che et al., 2016), though in *ex vivo* samples, CCR5 expression could be linked to the presence of leukocytes in the neoplastic areas (Sales et al., 2014). Natural ligands of CCR5 receptors include CCL3, CCL4, and RANTES (CCL5) (Oliveira et al., 2014; Vangelista and Vento, 2018) and Maraviroc has been shown to inhibit cytokine/chemokine effects at the CCR5 receptor (Dorr et al., 2005). In a preliminary experiment, Maraviroc concentrations of up to 20 μM were evaluated for potential negative effects on HeLa cells, which was ruled out (Supplementary Figure S2). Dorr et al. (2005) did not observe any negative effects on cell proliferation or cytotoxicity using Maraviroc concentrations up to 10 μM in PBMC and PM-1 cells. Concentration curve experiments evaluating inclusion morphology, size and number did not reveal changes indicative of chlamydial stress response or decreases in chlamydial inclusion size except for Maraviroc concentrations of 10 and 20 μM, which significantly reduced inclusion numbers compared to the control (Figure 5). To the author’s knowledge, there are no data available describing the interaction between Maraviroc and *C. trachomatis* or other chlamydial species. Sakthivel et al. (2008) attenuated the CCL5-CCR5 (RANTES-CCR5) axis by using anti-CCL5 antibodies in a mouse model with *C. muridarum* and revealed higher chlamydial loads in mice under anti-CCL5 treatment.

Maraviroc incubation of *C. trachomatis* infected HeLa cells led to a mild, but not significant increase of chlamydial infectivity at 10 μM and decrease at 20 μM. The reduction of chlamydial infectivity upon wIRA/VIS irradiation, however, was consistently observed after blocking the CCR5 receptor by Maraviroc (Figure 6). To conclude, the wIRA/VIS effect does not depend on uninhibited RANTES/CCR5 function.

Multiple studies, conducted in different models of chlamydial infection using wIRA alone or wIRA in combination with VIS irradiation, demonstrate that wIRA and wIRA/VIS have a stable and reproducible inhibitory effect on chlamydial infectivity (Marti et al., 2014, 2015; Rahn et al., 2016). Initial *in vitro* animal models using Vero cells and *C. pecorum*, as well as *in vitro* human models using HeLa cells and *C. trachomatis* Serovar E (genital model) or a combination of both, consistently demonstrated wIRA/VIS-dependent reduction of chlamydial inclusions and/or EBs (Marti et al., 2014, 2015). These previous experiments illustrated that wIRA and wIRA/VIS effects are independent of chlamydial strain or host cell line. Subsequently, primary cell lines (HCjE) were used in combination with *C. trachomatis* Serovar B to mimic an *in vitro* eye model, in which the same anti-chlamydial effects could be induced. Furthermore, irradiation of host cells (HCjE) prior to chlamydial infection led to a similar reduction of chlamydial infectivity as observed in previous studies, which indicates a wIRA-mediated impact on host cells possibly triggering a defense mechanism against chlamydial uptake (Rahn et al., 2016).

Al-Ahmad et al. (2013) were the first authors, who investigated the combination of photodynamic therapy and wIRA/VIS (antimicrobial photodynamic therapy [aPDT]). Excellent antimicrobial effects of aPDT have been demonstrated in multiple studies, leading to severe reductions of bacterial loads in multiple bacterial species and even in biofilms (Al-Ahmad et al., 2013, 2015, 2016; Karygianni et al., 2014). In another study, blue light with an emission peak at 460 nm was sufficient to severely reduce loads of *Aggregatibacter actinomycetemcomitans*, a periodontal pathogen (Cieplik et al., 2014). As a potential working mechanism, activation of endogenous photosensitizers and resulting antimicrobial effects were suspected (Cieplik et al., 2014). Since 460 nm is included in the wIRA/VIS spectrum, activation of endogenous photosensitizers by particular wavelengths might be involved in the working mechanism. Wavelength-specific effects on cellular structures have been suspected/reported by multiple authors, e.g., Karu et al. (2001) reviewed in Hoffmann (2007). Cytochrome c oxidase has recently been identified as a photo-acceptor for irradiation in the range of visible and near-infrared radiation (Passarella and Karu, 2014) and potential working mechanism of wIRA/VIS might involve changes in redox potential, changes in biochemical activity, production of reactive oxygen species and photodynamic actions as suspected by Karu (1999). Nonetheless, no working mechanism regarding antimicrobial effects of wIRA/VIS has been identified until now.

A previous study investigated cytokines as potential host cell factors being influenced by wIRA/VIS (Marti et al., 2014). In this study, we were able to gain a targeted insight into the host cellular immune response upon wIRA/VIS irradiation. We demonstrated that the reduction of chlamydial infectivity after wIRA/VIS irradiation is not abolished by pharmaceutical inhibition or gene silencing of host cell cytokines (IL-6, IL-8, and RANTES). Thus, factors other than host cell cytokine production must be involved in the working mechanism of wIRA/VIS.

## Author Contributions

NB initiated and supervised the study. Experiments were performed by JK, TP, and HM and mainly analyzed by JK. NB, CB, CL, and PT supported experimental procedures, analyses and interpretation, CB and CL additionally contributed to experimental design.

## Conflict of Interest Statement

The authors declare that the research was conducted in the absence of any commercial or financial relationships that could be construed as a potential conflict of interest.

## Supplementary Material

Figure S1 Gene silencing of IL-6, IL-8, and RANTES leads to sufficient reductions of mRNA levels over experimental time. HeLa cells were seeded, incubated for 24 h and transfected with siRNA for IL-6, IL-8, and RANTES gene silencing or mismatch siRNA. After an incubation of 5 h, media were replaced by HeLa growth medium. Sampling of monolayers for RNA isolation, reverse transcription and quantitative real time PCR was performed at 24, 48, 72, and 90 h post-transfection. Reduction of mRNA levels compared to mismatch-transfected samples and endogenous controls (actin beta) was evaluated by the 2^-ΔΔCT^ method and mRNA levels are expressed as percentage of mismatch controls. IL-6 and IL-8 samples were run in quadruplicates, RANTES samples in triplicates of two replicate samples.

Figure S2 Increasing ethanol and Celastrol concentrations lead to reduced cell viability, whereas Maraviroc concentrations up to 20 μM did not reduce cell viability. Cell viability assays were performed by seeding HeLa cells (5 × 10^4^ cells/well) followed by 24 h of incubation and replacement of incubation media by ethanol **(A)**, Celastrol **(B)** or Maraviroc **(C)** supplemented media at indicated concentrations. Cell viability was assessed at 12, 24, 36, and 45 h (ethanol) or 48 h (Celastrol and Maraviroc as represented by empty bars in Figures 2B,C). Cell viability is expressed as percentage of non-treated HeLa cells (**A** – control) or as percentage of ethanol-incubated HeLa cells (diluent-controls; **B,C**).
