# Supplementary figures and images for: Water Filtered Infrared A and Visible Light (wIRA/VIS) Irradiation Reduces Chlamydia trachomatis Infectivity Independent of Targeted Cytokine Inhibition
Source: Front Microbiol. 2018 Nov 15;9:2757. doi: 10.3389/fmicb.2018.02757 (PMC6262300; doi:10.3389/fmicb.2018.02757)

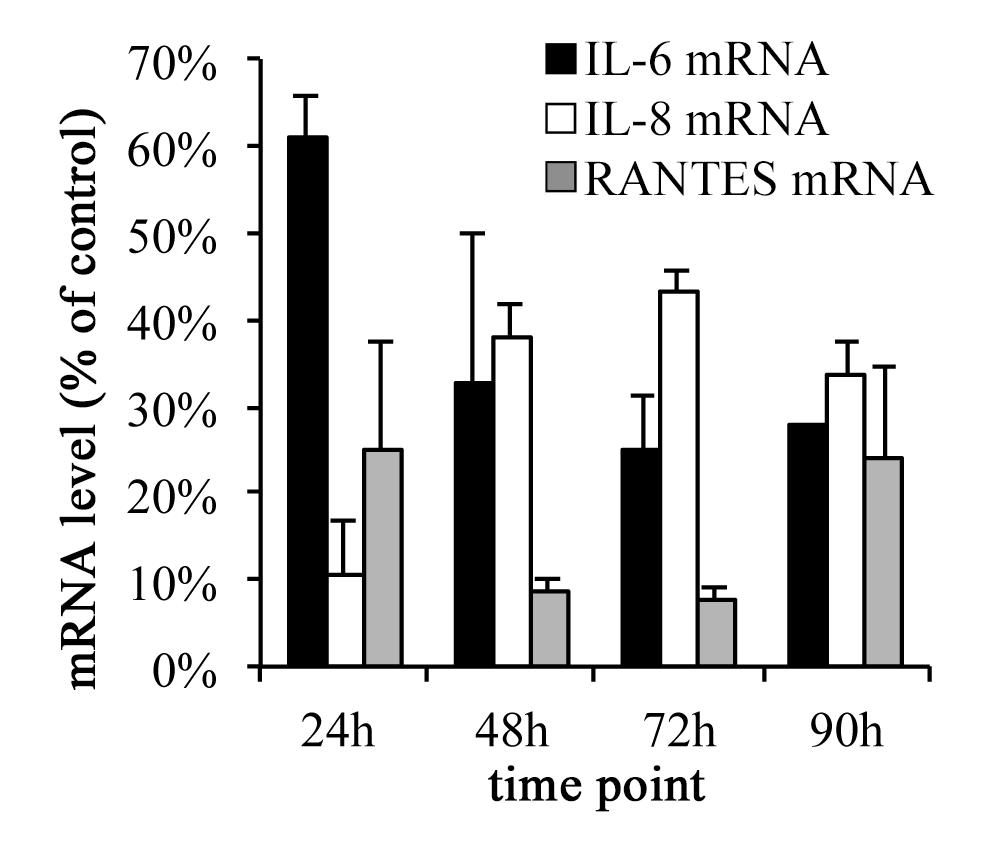

Supplement: Figure S1 — Gene silencing of IL-6, IL-8, and RANTES leads to sufficient reductions of mRNA levels over experimental time. HeLa cells were seeded, incubated for 24 h and transfected with siRNA for IL-6, IL-8, and RANTES gene silencing or mismatch siRNA. After an incubation of 5 h, media were replaced by HeLa growth medium. Sampling of monolayers for RNA isolation, reverse transcription and quantitative real time PCR was performed at 24, 48, 72, and 90 h post-transfection. Reduction of mRNA levels compared to mismatch-transfected samples and endogenous controls (actin beta) was evaluated by the 2-ΔΔCT method and mRNA levels are expressed as percentage of mismatch controls. IL-6 and IL-8 samples were run in quadruplicates, RANTES samples in triplicates of two replicate samples. [file Image_1.TIF]

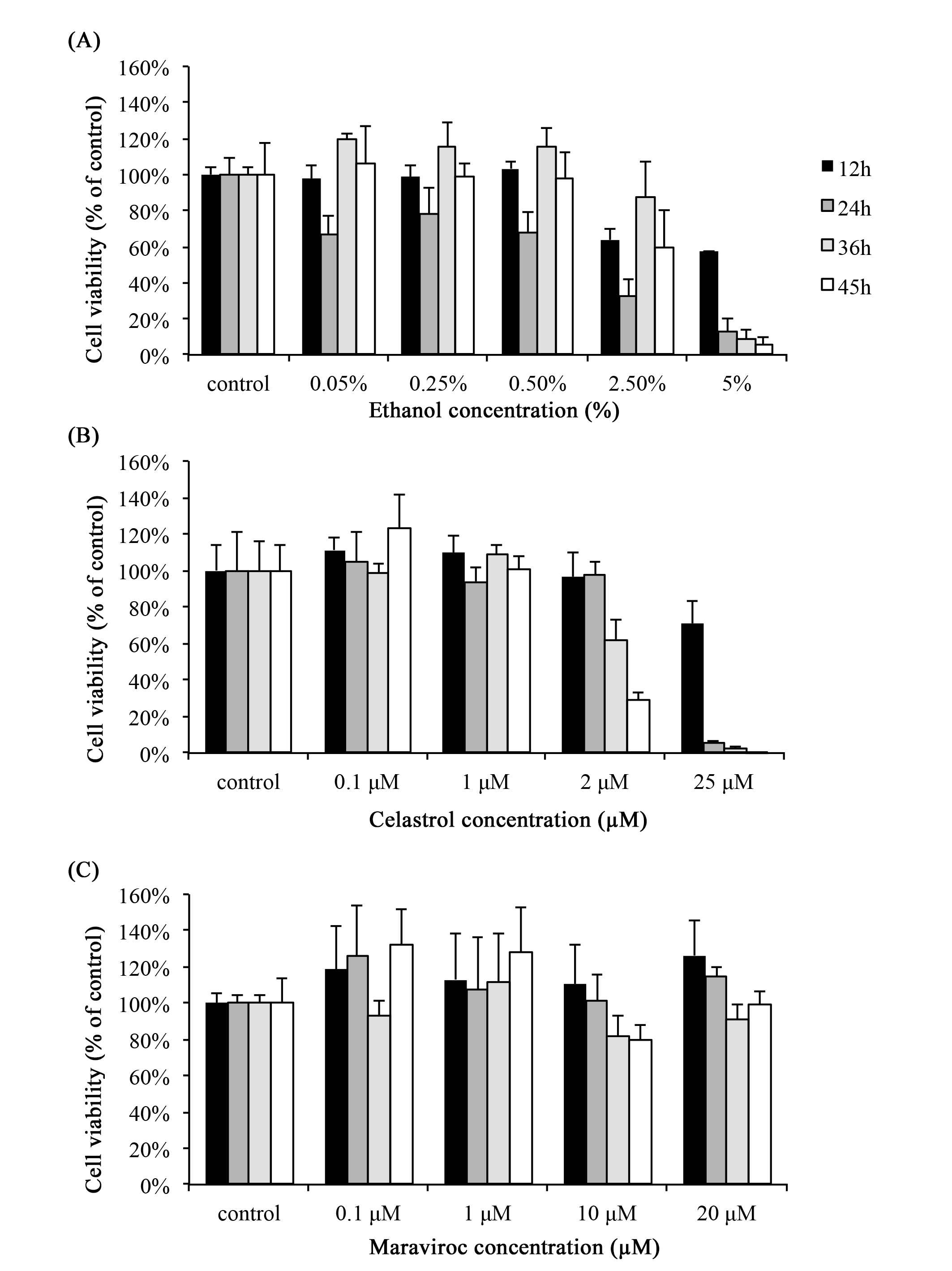

Supplement: Figure S2 — Increasing ethanol and Celastrol concentrations lead to reduced cell viability, whereas Maraviroc concentrations up to 20 μM did not reduce cell viability. Cell viability assays were performed by seeding HeLa cells (5 × 10^4^ cells/well) followed by 24 h of incubation and replacement of incubation media by ethanol (A), Celastrol (B) or Maraviroc (C) supplemented media at indicated concentrations. Cell viability was assessed at 12, 24, 36, and 45 h (ethanol) or 48 h (Celastrol and Maraviroc as represented by empty bars in Figures 2B,C). Cell viability is expressed as percentage of non-treated HeLa cells (A – control) or as percentage of ethanol-incubated HeLa cells (diluent-controls; B,C). [file Image_2.TIF]
